# Treatment of Anæmia by Arsenite of Copper

**Published:** 1892-04

**Authors:** 


### TREATMENT OF ANÆMIA BY ARSENITE OF COPPER.

---

Dr. H. A. Hare, in the *Therapeutic Gazette*, highly commends the arsenite of copper in functional anæmia. He considers it one of the remedies which should always be tried, and second only to iron and arsenious acid. Copper closely resembles arsenic, both physiologically and therapeutically, and may be considered under certain conditions a normal constituent of the blood of man. It has been shown experimentally that the double salts of copper are a direct stimulant to the heart and walls of the capillaries, and that small doses have a tonic effect on the nervous system.

Anæmia is only a symptom of organic or functional disorder. Organic anæmia is practically incurable, and it is only the functional forms of the disease that afford us opportunities of promoting a cure. And it is in just these cases that arsenite of copper is indicated, particularly if the gastro-intestinal canal is out of order.

Acting, as does arsenic, as a stimulant to mucous membranes all over the body, in addition to its stimulant influence on nutrition, it tends to prevent disorders of the digestive mucous membrane, and so renders perfect secretion and absorption possible, preventing the auto-intoxication of the patient from the fermentation and decomposition changes in the contents of the stomach and bowel. Happily joined to this, the copper adds tone to the system, and promotes assimilation and the production of muscular tissue. To such an extent does this drug act as an alterative that Luton has used the phosphate of copper as a remedy of peculiar virtue in scrofulosis and tuberculosis, so that we may consider the drug as one possessing a peculiar influence similar to that possessed by minute doses of bichloride of mercury.

Acting in the belief that arsenite of copper would form a useful combination in the treatment of anæmia and debility, the writer has tried it in a number of cases with very encouraging results. Under these circumstances the digestion improves, the color becomes more like the normal, and either by a direct effect on nutrition or on digestion, or on both, the patients progressed rapidly towards health, provided of course, that the anæmia was functional and not organic in origin.

In the dose of $\frac{1}{50}$ or $\frac{1}{25}$ of a grain, arsenite of copper will, I think, often prove of service, if given three times a day after meals, and from its combination may prove to be superior to Fowler's solution not only in anæmia, but also in chorea and similar nervous ailments.

